# Beraprost sodium attenuates the development of myocardial fibrosis after myocardial infarction by regulating GSK‐3β expression in rats

**DOI:** 10.1002/iid3.1050

**Published:** 2023-11-09

**Authors:** Xing‐Xing Li, Yun‐Zhe Wang, Chuang Liu, Guo‐Wei Fu, Jun Li, Jin‐Ying Zhang

**Affiliations:** ^1^ Department of Extracorporeal Life Support Center The First Affiliated Hospital of Zhengzhou University Zhengzhou China; ^2^ Department of Cardiology The First Affiliated Hospital of Zhengzhou University Zhengzhou China; ^3^ Henan Province′s Key Laboratory of Cardiac Injury and Repair Zhengzhou China; ^4^ Henan Province Clinical Research Center for Cardiovascular Diseases Zhengzhou China

**Keywords:** beraprost sodium, cAMP/p‐CREB, myocardial fibrosis, myocardial infarction, YBX1/GSK‐3β

## Abstract

**Objective:**

The aim of this study was to elucidate the mechanism of beraprost sodium (BPS) in the intervention of myocardial fibrosis after myocardial infarction (MI) through glycogen synthase kinase‐3β (GSK‐3β) and to provide new ideas for intervention in myocardial fibrosis.

**Materials and Methods:**

MI model rats given BPS and cardiac fibroblasts (CFs) treated with BPS and TGF‐β. HE staining and Masson staining were used to detect the pathological changes of myocardial tissue. Fibrotic markers were detected by immunohistochemical staining. The expressions of GSK‐3β, cAMP response element binding protein (CREB), and p‐CREB were analyzed by qPCR and western blot analysis. EDU staining was used to detect the proliferation of CFs. The promoter activity of GSK‐3β was detected by luciferase assay. Chromatin immunoprecipitation assay was used to detect the binding levels of GSK‐3β promoter and Y‐box binding protein 1 (YBX1). The levels of intracellular cyclic adenosine monophosphate (cAMP) were analyzed by enzyme‐linked immunosorbent assay (ELISA).

**Results:**

After operation, BPS improved myocardial fibrosis and upregulated GSK‐3β protein expression in male SD rats. BPS can down‐regulate α‐smooth muscle actin (α‐SMA) level and up‐regulate GSK‐3β protein expression in CFs after TGF‐β stimulation. Furthermore, GSK‐3β knockdown can reverse the effect of BPS on TGF‐β‐activated CFs, enhance α‐SMA expression, and promote the proliferation of CFs. BPS could regulate GSK‐3β expression by promoting the binding of GSK‐3β promoter to YBX1. BPS induced upregulation of p‐CREB and cAMP, resulting in reduced fibrosis, which was reversed by the knockdown of GSK‐3β or prostaglandin receptor (IPR) antagonists.

**Conclusion:**

BPS treatment increased the binding of YBX1 to the GSK‐3β promoter, and GSK‐3β protein expression was upregulated, which further caused the upregulation of p‐CREB and cAMP, and finally inhibited myocardial fibrosis.

## Graphical abstract

This study found that for the first time GSK‐3β are the target of BPS. In this study, it was found for the first time that BPS regulated the expression of GSK‐3β by interfering with the binding of GSK‐3β promoter to YBX1.

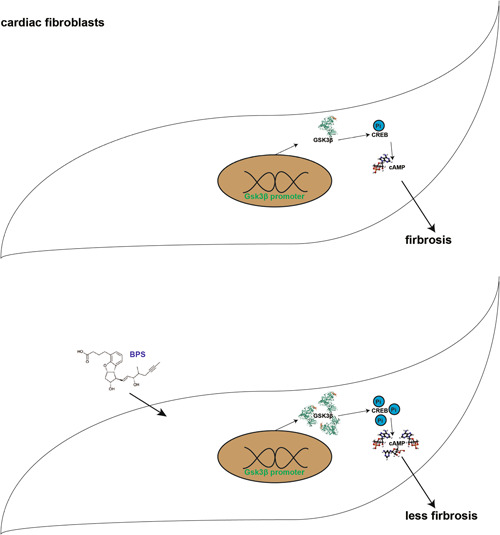

## 1 INTRODUCTION

As a common clinical disease, myocardial infarction (MI) is associated with myocardial ischemia, leading to arrhythmia, expansion of infarct size, and ultimately ventricular systolic dysfunction.^1^, ^2^ MI has a tremendous negative impact on human health and has become a significant challenge in clinical practice. However, the pathogenesis of MI involves a complex and multifactorial process, so effective treatments are still rare. To explore a novel and effective treatment strategy for MI, in‐depth analysis of the relevant drug treatment mechanisms is essential. Myocardial fibrosis is the primary pathological process after MI, characterized by the proliferation of myocardial interstitial fibroblasts and excessive deposition of collagen fibers.^3^, ^4^ Therefore, clarifying the mechanism and influencing factors of myocardial fibrosis provides a new perspective for the clinical treatment of MI in the future.

Beraprost sodium (BPS), as a prostaglandin analog, is used for cardiovascular diseases such as heart failure.^5^ The combination of sildenafil and BPS has been reported to inhibit arrhythmia.^6^ Studies have shown that BPS can improve cardiac function in rats with MI, and significantly delay the growth rate of cardiac fibroblasts and reduce collagen expression in vivo.^7^, ^8^ Zmajkovicova and colleagues found that BPS delayed TGF‐β1‐induced lung fibroblast to myofibroblast.^9^ Long‐term administration of BPS can reduce myocardial fibrosis caused by hypertensive cardiac hypertrophy.^10^ This experiment will verify the effect of BPS on myocardial fibrosis after MI.

Notably, Glycogen synthase kinase‐3β (GSK‐3β) was found to be involved in the remodeling process after MI in rats.^11^, ^12^ GSK‐3β knockdown in fibroblasts can aggravate myocardial fibrosis and adverse ventricular remodeling after MI, and GSK‐3β deletion in cardiomyocytes leads to ventricular obstruction in mice.^12^, ^13^ GSK‐3β expression was downregulated after MI in rats induced by the left colic artery, and a similar phenomenon was observed in rats with heart failure induced by streptozotocin.^14^, ^15^ GSK‐3β was upregulated, and collagen, a marker of fibrosis, was downregulated after TGF‐β inhibition in renal cells.^16^ At the same time, it was reported that the upregulation of prostaglandin E2, a similar substance to BPS, caused the upregulation of GSK‐3β in B cells.^17^ In hepatic ischemia‐reperfusion, GSK‐3β expression is activated after the use of prostaglandin receptor (IPR) agonists.^18^ Therefore, we inferred BPS affected GSK‐3β expression in MI.

Preliminary experiments demonstrated that GSK‐3β was upregulated in cardiac fibroblasts (CFs) of MI rats after BPS administration. However, the molecular mechanism of how BPS regulates GSK‐3β expression in myocardial fibrosis is worth further investigation.

## 2 MATERIALS AND METHODS

### 2.1 Establishment of MI model in rats

Eighteen SPF SD male rats (weighing 185–215 g, aged 6–7 weeks, Chang Zhou Cavens Laboratory Animal Co., Ltd) were kept in a suitable environment with room temperature (22 ± 3)°C, humidity 50%–70%, 12/12 h light/dark cycle, standard diet, free drinking and eating. After fed adaptively for 7 days, the rats were used for experimental study. The rats were anesthetized by intraperitoneally injecting 1% sodium pentobarbital (50 mg/kg) and then fixed in supine position. The chest cavity was transected at the fourth intercostal region of the left chest to expose the heart. In the MI model group or BPS group, the left anterior descending coronary artery was ligated with 6–0 line. In the sham group, the left anterior descending coronary artery was only transected without ligation. BPS (15 μg/kg/day, Beijing Tide Pharmaceutical Co., Ltd.) was injected subcutaneously into the BPS group immediately after coronary artery ligation. The same volume of saline was injected in the sham group or MI group for 4 weeks. The rats were killed 1 week after the last dose, and then heart tissues were collected.^7^ In each group, three tissues were used for homogenization and another three for dehydrated sections. Animal experiments in this study are approved by the Ethics Committee of The First Affiliated Hospital, Zhengzhou University (Approved No.: 2021060301).

### 2.2 Hematoxylin‐eosin (HE) and Masson staining

The left ventricular myocardial tissues were fixed in 4% paraformaldehyde (Beyotime) and embedded in paraffin after conventional treatment. Paraffin Section (5 μm) were prepared and stained according to the HE kit instructions (Beyotime). The histopathological changes were observed under the light microscope (Leica).

After dewaxing and hydration, the paraffin sections were stained with hematoxylin and Ponceau S according to the instructions (Beyotime), treated with phosphomolybdic acid for about 3 min, and counterstained with aniline blue for 5 min. After staining, the cells were washed, dehydrated, and transparent with xylene. After sealing the slices, the results were observed under the light microscope, in which cardiomyocytes were stained red, and collagen fibers were stained blue.

### 2.3 Immunohistochemical staining

The 5 μm paraffin sections were dewaxed to water, and then sodium citrate buffer (10 nmol/L, pH 6.0) was added for antigen heat repair. Endogenous peroxidase was inactivated in 3% H_2_O_2_, and blocked with 5% bovine serum albumin (Beijing Solarbio Science & Technology Co., Ltd) for 1 h at room temperature. The primary antibodies α‐smooth muscle actin (α‐SMA, ab7817; abcam) and collagen Ⅰ (ab138492; abcam) were added and incubated at 4°C overnight. Next, the biotin‐labeled secondary antibody was added and incubated at 37°C for 30 min, followed by strept actividin‐biotin complex (SABC) reagent (BOSTER Biological Technology Co. Ltd) for 20 min, followed by 3,3'‐diaminobenzidine (DAB) color development, hematoxylin counterstaining, dehydration, and sealing. The Image were observed and taken under a light microscope.

### 2.4 Western blot

Cells in each group were harvested and lysed, and protein concentrations were detected by BCA medthod (Sangon Biotech [Shanghai] Co., Ltd). Twenty micrograms of protein was loaded and placed in sodium dodecyl sulfate polyacrylamide gel electrophoresis (SDS‐PAGE) for electrophoresis to isolate the protein. The separated protein was transferred to PVDF membrane (Millipore), and blocked in TBST (containing 50 g/L skim milk powder) for 1–2 h at room temperature. GSK‐3β (ab280376; abcam) and p‐cAMP response element binding protein (p‐CREB, ab32096, abcam; CREB, ab32515, abcam) antibodies were added and incubated overnight at 4°C. The next day, PVDF membrane was washed three times with TBST. The secondary antibody was diluted with TBST (containing 50 g/L skim milk powder) and incubated for 1 h. After the PVDF membrane was washed three times with TBST, the Image was developed by a gel imager (Invitrogen).

### 2.5 Culture and treatment of primary CFs

After 1–3 days SD rats were immersed in 75% ethanol for disinfection, the hearts were removed, cut and digested with 1% trypsin and 0.125% collagenase type II mixture (Beijing Solarbio Science & Technology Co., Ltd). After centrifugation, fibroblasts and cardiomyocytes were seeded in cell culture flaps and separated according to the adhesion time. Fibroblasts adherent 1.5 h before cultured in the dulbecco's modified eagle media (Gibco) with 100 U/mL penicillin, 100 mg/mL streptomycin and 10% fetal bovine serum (Gibco) in an incubator with 5% CO_2_ at 37°C. Culture medium was changed every 48 h.^19^ The cells were treated with TGF‐β (10 ng/mL),^20^ BPS (0, 5, 10, 20 μmol/L)^8^ and CAY10441 (1 μmol/L)^21^ for 24 h when the cell density reached 60%, and grouped into blank, TGF‐β, TGF‐β+BPS, TGF‐β+BPS+CAY groups.

### 2.6 Cell transfection

CFs in the logarithmic growth phase were collected, digested with trypsin, and seeded into 96‐well plates with 5 × 10^4^ cells/well. CFs were divided into TGF‐β, TGF‐β+BPS, TGF‐β+BPS+si‐NC, TGF‐β+BPS+si‐GSK‐3β, TGF‐β+BPS+si‐Y‐box binding protein 1 (YBX1) groups. Cells in TGF‐β and TGF‐β+BPS groups were not transfected. Cells in TGF‐β+BPS+si‐GSK‐3β, TGF‐β+BPS+si‐YBX1, and TGF‐β+BPS+si‐NC groups were transfected with GSK‐3β siRNA (Shanghai Genechem) and YBX1 siRNA (Shanghai Genechem) and siRNA NC (Shanghai Genechem), respectively. Transfection was performed using Lipfeclamine 2000 transfection kit (Thermo Fisher). After 24 h, cells were cultured with TGF‐β and/or BPS, and divided into OE‐YBX1, OE‐NC, si‐YBX1, and si‐NC groups.

### 2.7 CCK‐8 assay

The cells with 80% cell confluence were digested by trypsin and seeded into a 96‐well plate with 2 × 10^3^ cells/well. When the confluence reached 80%, BPS was added at a concentration of 5, 10, and 20 μmol/L, respectively. After 24 h, 10 μL of CCK‐8 solution (Beyotime) was added, and then incubated for 3 h. The absorbance at 450 nm was measured by a microplate reader (Thermo Scientific), and the cell viability was calculated to determine the concentration of BPS in subsequent experiments.

### 2.8 Immunofluorescence assay

After CFs in each group were cultured for 24 h, the culture medium was aspirated and rinsed with cold PBS for three times. The samples were freshly prepared and fixed with 4% paraformaldehyde for 30 min, and then rinsed with PBS for three times at room temperature. After treatment with 0.3% Triton X‐100 for 30 min, the cells were rinsed with PBS for three times and blocked with goat serum working solution for 60 min. α‐SMA primary antibody was added and incubated overnight at 4°C. Next, the samples incubated with fluorescein isothiocyanate (FITC) labeled secondary antibody for 1 h at room temperature. After rinsing with PBS and staining with DAPI, the staining results were observed under a fluorescence microscope (Leica).

### 2.9 5‐ethynyl‐2'‐deoxyuridine (EDU) staining

CFs were seeded in 6‐well plates, cultured for 24 h, and then replaced with serum‐free medium for 24 h. CFs were incubated with EdU working solution (final concentration: 50 μmol/L) for 5 h according to the kit instructions (Beyotime), the medium was removed, and 4% paraformaldehyde was added for 15 min at room temperature. The fixation solution was removed and the cells were washed three times with washing solution. The washing solution was removed, and the cells were incubated with 0.3% Triton X‐100 solution at room temperature for 15 min. The cells were washed twice with the washing solution. Five hundred micrograms of reaction solution was added and incubated for 30 min at room temperature under light. The fluorescence intensity was measured on a microplate reader.

### 2.10 Quantitative real‐time polymerase chain reaction (qPCR)

Total RNA was extracted according to Trizol one‐step extraction kit (Wuhan Servicebio Technology CO., LTD) and the concentration and purity of RNA were detected using NanoDrop2000 (Thermo Scientific). Three hundred nanograms of total RNA was reverse‐transcribed into cDNA according to the procedure of the first‐strand DNA synthesis kit (Thermo Scientific). Ten nanograms of cDNA was used as template, and GSK‐3β level was detected by SYBR‐Green Ⅰ (TaKaRa) in 10 μL qPCR reaction system. The reaction procedure was 95°C for 3 min, 95°C for 5 s, 60°C for 30 s, and 35 cycles of amplification. The relative expression of GSK‐3β was calculated by $2^{‐∆∆C_{t}}$ method with GAPDH as the internal reference.

### 2.11 Luciferase assay

The plasmid of 1 kb, including 151–666 bp upstream of GSK‐3β transcription start site, was cloned to renilla luciferase‐expression plasmid. After being transfected with GSK‐3β reporter gene vector according to the procedure of Lipofectamine 2000 transfection kit (Thermo Fisher), CFs were cultured with TGF‐β and/or BPS. CFs were co‐transfected with GSK‐3β reporter gene vector and OE‐YBX1, OE‐NC, si‐YBX1, or si‐NC of Lipofectamine 2000. After 48 h of culture in normal medium, the cells in OE‐YBX1, OE‐NC, si‐YBX1, and si‐NC groups were lysed, and the activities of firefly and Renilla luciferases were detected by using a dual‐luciferase reporter assay system.

### 2.12 Chromatin immunoprecipitation assay (ChIP)

CFs were cross‐linked with 1% formaldehyde for 10 min, the formaldehyde was neutralized with glycine. The cells were washed with PBS for three times. The samples were incubated at 37°C for 20 min. After the cells were broken by ultrasound and centrifuged, the supernatant was collected and divided into YBX1 and negative control groups. Anti‐YBX1 and IgG were added and incubated overnight at 4°C. After antibodies and YBX1‐DNA complex were combined, protein G magnetic beads were added, and the precipitated complex was eluted. After de‐crosslinking, the DNA fragments were purified and enriched by DNA purification column, and the GSK‐3β promoter DNA was analyzed by qPCR.

### 2.13 Enzyme‐linked immunosorbent assay (ELISA)

After CFs were lysed, neutralization buffer and acetylation reagent were added. After shaking and incubation, the cells were transferred to a 96‐well plate, and followed the instructions of the Direct Cyclic AMP EIA kit (Enzo).^22^ The absorbance value at 450 nm was analyzed with a microplate plate and compared with the cyclic adenosine monophosphate (cAMP) standard curve to calculate cAMP concentration.

### 2.14 Statistical analyses

The cell experiment was repeated three times. GraphPad Prism8.0.2 was used for statistical analysis, and the data was expressed as mean ± S. The Anderson–Darling test, Shapiro–Wilk test, D'Agostino & Pearson's test or Kolmogorov–Smirnov test was used to analyze whether the data were normally distributed. *F* test was used to test the homogeneity of variance for the two groups of data with normal distribution. If *p* value of *F* test is ≥.05, the two‐tailed unpaired *t* test is used for data comparison. ANOVA test was used to test the homogeneity of variance for the multi‐group of data with normal distribution. *p* value of Brown–Forsythe test and Welch's ANOVA test is <.05, which means that the variance is nonhomogeneity. *p* < .05 was considered as statistically significant difference.

## 3 RESULTS

### 3.1 Expression of GSK‐3β and myocardial histomorphology in rats with MI after BPS administration

**Figure 1:**
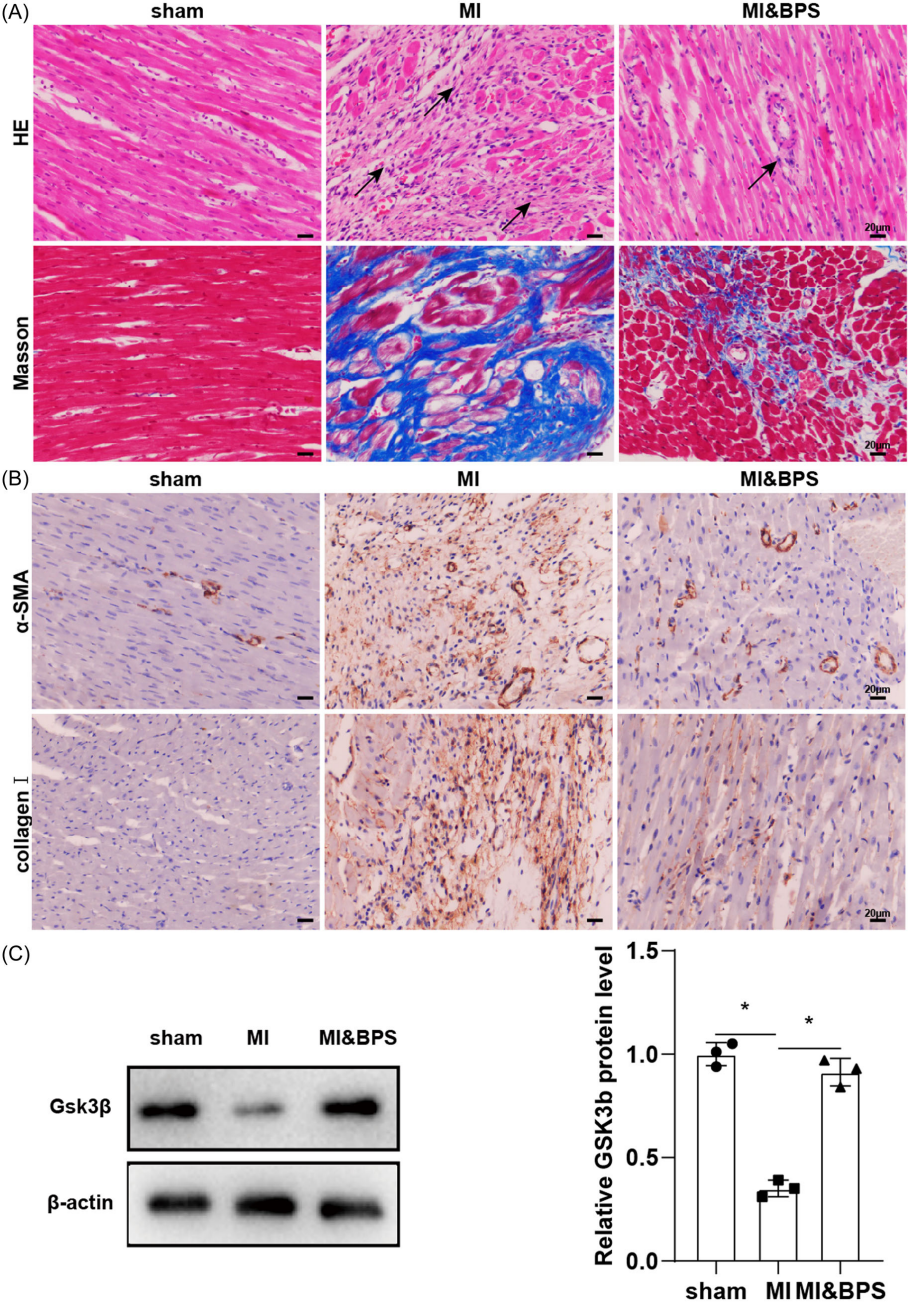
Expression of GSK‐3β and myocardial histomorphology in rats with MI after BPS administration. The rats were divided into sham group, MI group and MI&BPS group. *n* = 6/group. (A) The pathological changes of myocardial tissue were detected by HE staining. Masson staining was used to detect the degree of myocardial fibrosis. (B) Fibrotic markers α‐SMA and Collagen I were detected by immunohistochemical staining. (C) The protein expressions of GSK‐3β in myocardial tissue were analyzed by Western blotting. *p ＜ .05 versus Sham group or MI group. BPS, beraprost sodium; MI, myocardial infarction.

In the HE staining results of myocardial tissue sections, the cardiomyocytes were arranged regularly in the sham group and showed normal size and shape, while in the MI group, the arrangement of cardiomyocytes were disordered, the collagen increased significantly, and the cell volume increased significantly (Figure 1A). Compared with MI group, the histopathological changes of BPS group were alleviated (Figure 1A). Masson staining showed that the myocardial cells in the sham group were arranged neatly and the size was normal (Figure 1A). In the MI group, the area of collagen fibers was increased, the cell size was different, and the structure disappeared (Figure 1A). Compared with MI group, the area of collagen fibers was decreased in the BPS group (Figure 1A). In addition, the α‐SMA and collagenⅠ positive cells in the MI group were significantly increased when compared with the sham group (Figure 1B). The levels of α‐SMA and CollagenⅠ in BPS group were significantly lower than those in MI group (Figure 1B). BPS plays a role in improving myocardial fibrosis in MI rats, which was consistent with the results of previous studies.^8^ Western blotting showed that GSK‐3β protein expression in the myocardial tissues of MI group was decreased, and then increased after BPS treatment (Figure 1C). Next, we further investigated whether GSK‐3β is a target of BPS in vitro.

### 3.2 BPS treatment interferes with GSK‐3β expression in CFs in vitro

**Figure 2:**
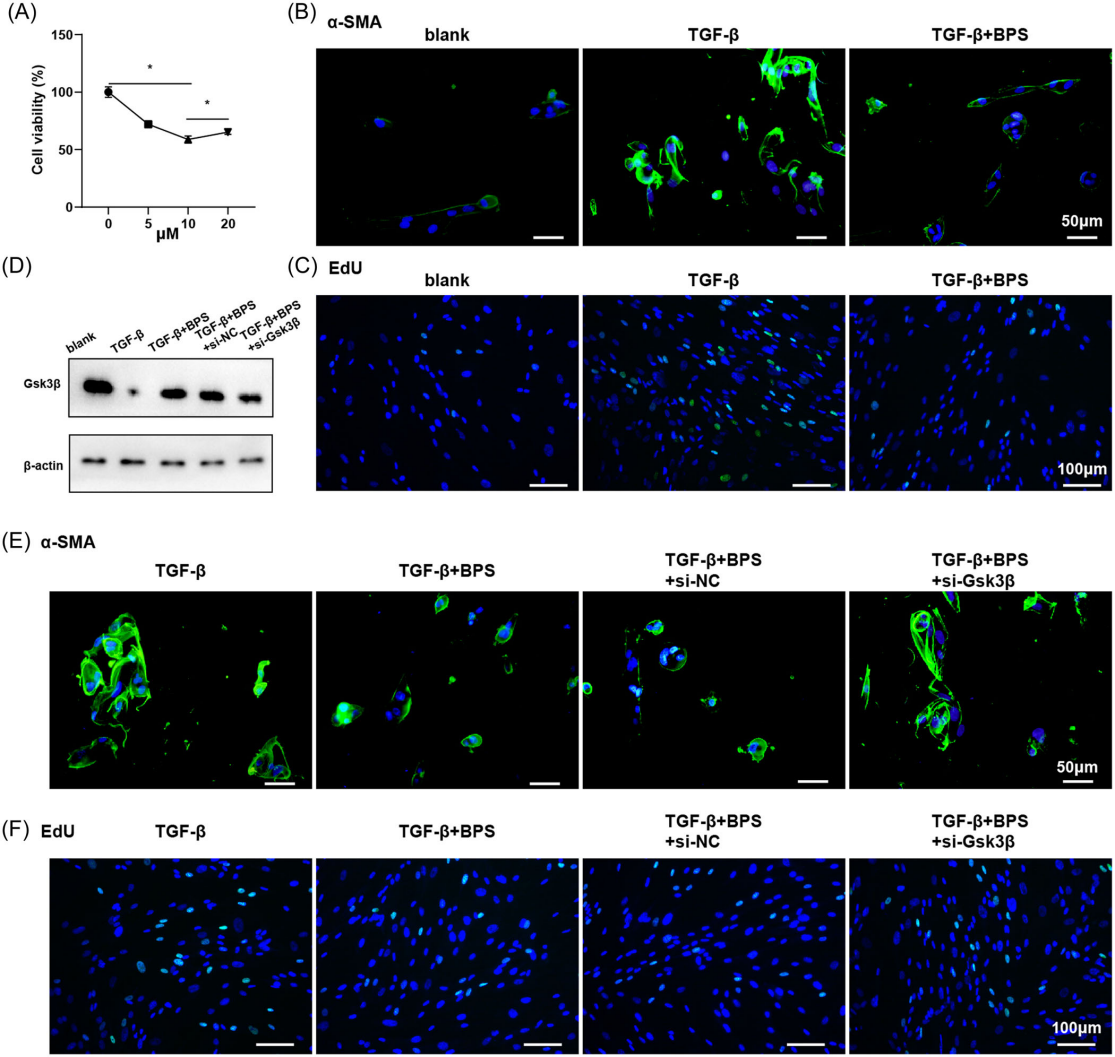
BPS treatment interferes with GSK‐3β expression in CFs in vitro. (A) CFs were treated with TGF‐β (10 ng/mL) and BPS (0, 5, 10, and 20 μmol/L). Cell viability was detected by CCK‐8 assay. CFs were grouped into blank, TGF‐β, TGF‐β+BPS (10 μmol/L). *p  ＜ .05. (B) α‐SMA level was detected by immunofluorescence staining. (C) EDU was used to detect the proliferation of CFs. CFs were grouped into blank, TGF‐β, TGF‐β+BPS, TGF‐β+BPS+si‐NC, TGF‐β+BPS+si‐GSK‐3β. (D) Western blot was used to detect the expressions of GSK‐3β. (E) α‐SMA level was detected by immunofluorescence staining. (F) EDU was used to detect the proliferation of CFs. BPS, beraprost sodium; CFs, cardiac fibroblasts.

To investigate the level of GSK‐3β in activated CFs, primary CFs were stimulated with TGF‐β to establish a cellular fibrosis model. CCK‐8 assay showed that the inhibitory effect was strongest at 10 μM (Figure 2A). Next, 10 μM TGF‐β was used to treat CFs. Immunofluorescence assay showed that BPS could down‐regulate the level of α‐SMA in TGF‐β‐activated CFs, which was consistent with the in vivo results (Figure 2B). EDU assay showed that TGF‐β treatment promoted CFs proliferation, while BPS could reverse this effect (Figure 2C). CFs were infected with siRNA lentivirus of GSK‐3β, and divided into TGF‐β, TGF‐β+BPS, TGF‐β+BPS+si‐NC, TGF‐β+BPS+si‐GSK‐3β. Western blotting showed that BPS treatment enhanced GSK‐3β protein expression, which was reversed by further transfection of si‐GSK‐3β, indicating successful plasmid construction (Figure 2D). Immunofluorescence detection of α‐SMA changes showed that downregulation of GSK‐3β could reverse the effect of BPS on α‐SMA and enhance the expression of α‐SMA (Figure 2E). EDU assay showed that knockdown of GSK‐3β promoted the proliferation of TGF‐β‐activated CFs, and the effect of BPS on α‐SMA almost disappeared (Figure 2F). It is worth noting that BPS had no significant effect on CFs proliferation without TGF‐β stimulation (Supporting Information: Figure 1). The above results showed that BPS regulates myocardial fibrosis by targeting GSK‐3β upregulation.

### 3.3 BPS treatment upregulates GSK‐3β promoter transcription in vitro

**Figure 3:**
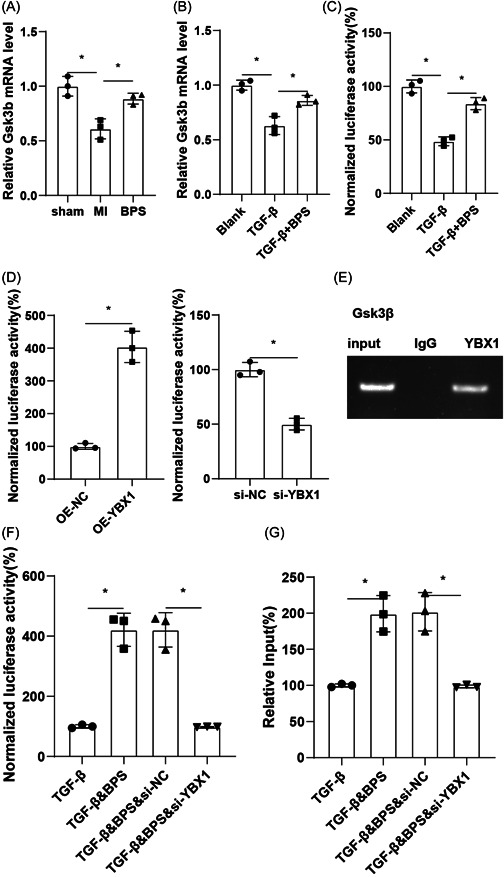
BPS treatment upregulates GSK‐3β promoter transcription in vitro. (A) qPCR was used to detect the mRNA levels of GSK‐3β in the myocardial tissues of sham group, MI group and MI&BPS group. *p ＜ .05 versus Sham group or MI group. (B) qPCR was used to detect the mRNA levels of GSK‐3β in CFs of blank group, TGF‐β group and TGF‐β+BPS group. (C) The promoter fluorescence reporter gene of GSK‐3β was constructed, and the promoter activity of GSK‐3β in CFs of blank, TGF‐β and TGF‐β+BPS groups was detected by luciferase assay. *p  ＜  .05 versus blank group or TGF‐β group. (D&E) The promoter activity of GSK‐3β in CFs transfected with OE‐YBX1, OE‐NC, si‐YBX1, or si‐NC were detected by luciferase assay. *p ＜ .05 versus OE‐NC group or si‐NC group. (E) ChIP assay was used to detect the binding levels of GSK‐3β promoter and YBX1. CFs were grouped into TGF‐β, TGF‐β+BPS, TGF‐β+BPS+si‐NC, TGF‐β+BPS+si‐YBX1. (F) The promoter activity of GSK‐3β was detected by luciferase assay. (G) ChIP assay was used to detect the binding levels of GSK‐3β promoter and YBX1 in CFs. *p ＜ .05 versus TGF‐β group or TGF‐β+BPS+si‐NC. CFs, cardiac fibroblasts; MI, myocardial infarction.

The above experiments only detected the changes in the GSK‐3β protein expression. Next, we explored whether BPS had effects on the transcription level of GSK‐3β. QPCR showed that the mRNA level of GSK‐3β increased after administration (Figure 3A). The mRNA levels of GSK‐3β in Blank, TGF‐β and TGF‐β+BPS groups showed the same results (Figure 3B). Figure 3C results showed that BPS could enhance the promoter activity of GSK‐3β. Furthermore, we verified the transcriptional regulation of GSK‐3β by YBX1 in CFs, and found that the promoter activity of GSK‐3β was positively regulated by YBX1 (Figure 3D). ChIP assay verified that the promoter of GSK‐3β binds to YBX1 in CFs (Figure 3E). To investigate the effect of BPS on the binding level of GSK‐3β promoter and YBX1, CFs were grouped into TGF‐β, TGF‐β+BPS+si‐NC, TGF‐β+BPS+si‐YBX1 groups. Luciferase reporter assay showed that downregulation of YBX1 could reverse the upregulation of GSK‐3β promoter activity caused by BPS (Figure 3F). Furthermore, the binding level of GSK‐3β promoter and YBX1 in CFs was upregulated after the addition of BPS, and si‐YBX1 reversed this effect (Figure 3G).

### 3.4 BPS interferes with myocardial fibrosis through cAMP signaling pathway

**Figure 4:**
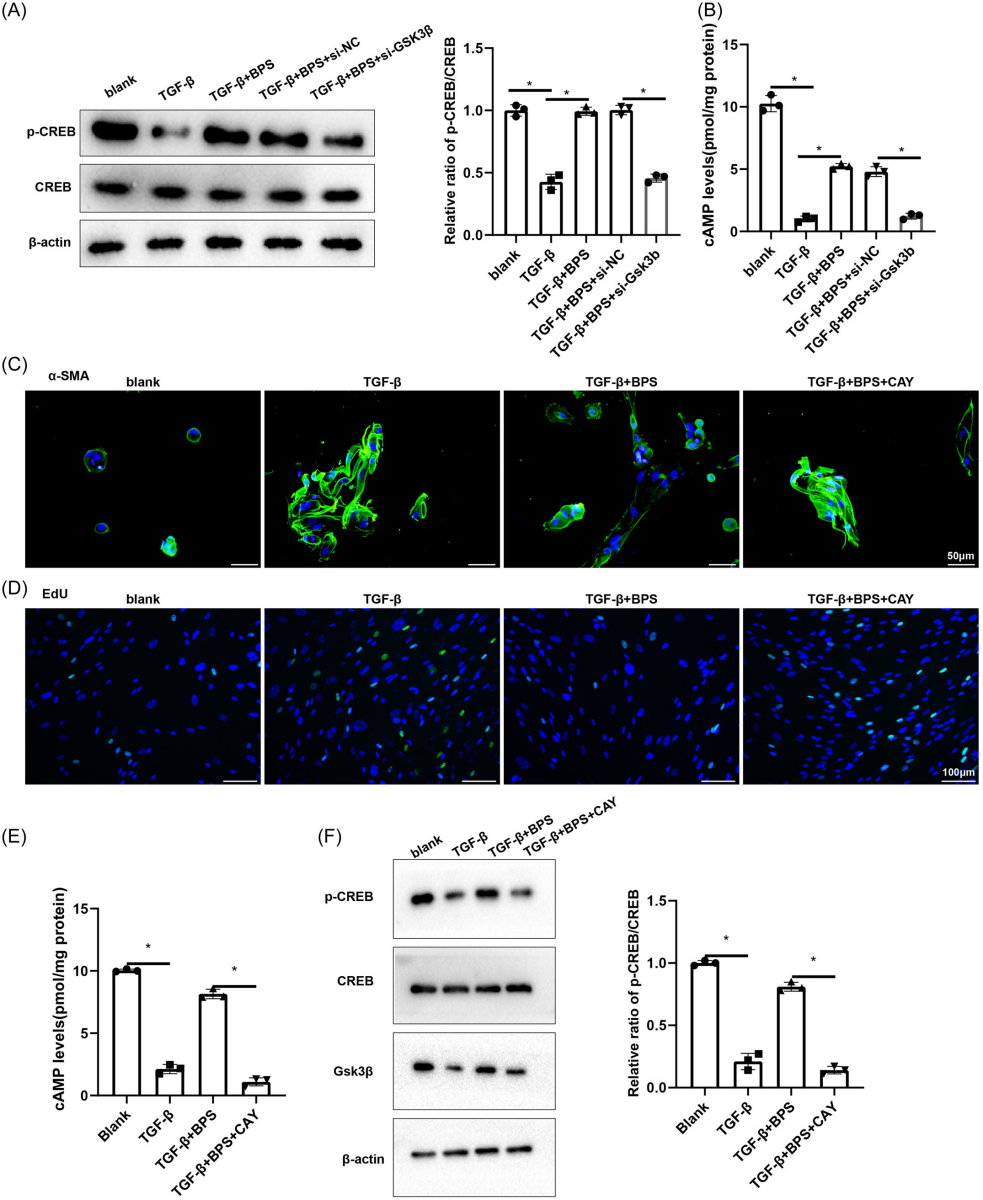
BPS treatment interferes with myocardial fibrosis through activation of prostacyclin receptor. CFs were grouped into TGF‐β, TGF‐β+BPS, TGF‐β+BPS+si‐NC, TGF‐β+BPS+si‐GSK‐3β. (A) Western blotting was used to detect the expressions of p‐CREB and CREB. The relative ratio of p‐CREB/CREB was detected. (B) The levels of intracellular cAMP in each group were analyzed by ELISA. *p  ＜  .05 versus TGF‐β or TGF‐β+BPS+si‐NC. CFs were grouped into blank, TGF‐β, TGF‐β+BPS, TGF‐β+BPS+CAY (1 μM). (C) α‐SMA level was detected by immunofluorescence staining. (D) EDU was used to detect the proliferation of CFs. (E) The levels of intracellular cAMP were analyzed by ELISA. (F) Western blotting was used to detect the expressions of GSK‐3β, p‐CREB, and CREB. The relative ratio of p‐CREB/CREB was detected. *p  ＜  .05 versus blank or TGF‐β+BPS+CAY. (G) Molecular mechanism diagram of BPS regulating the development of myocardial fibrosis after MI. BPS, beraprost sodium; MI, myocardial infarction.

We next investigated whether BPS acts on myocardial fibrosis through YBX1/GSK‐3β ultimately affecting cAMP signaling pathway. CFs were grouped into TGF‐β, TGF‐β+BPS, TGF‐β+BPS+si‐NC, TGF‐β+BPS+si‐GSK‐3β groups. The relative ratio of p‐CREB/CREB showed that BPS could enhance p‐CREB protein expression, and the knockdown of GSK‐3β revised this effect (Figure 4A). The levels of intracellular cAMP in each group were analyzed, and the results showed that the effect of BPS on cAMP decreased after knockdown of GSK‐3β (Figure 4B). Next, prostacyclin receptor (IPR) antagonist CAY (1 μM) was used to test whether the effect of BPS on GSK‐3β was related to cAMP signaling pathway. CFs were grouped into blank, TGF‐β, TGF‐β+BPS, TGF‐β+BPS+CAY groups. The results of IF and EDU assays showed that the degree of fibrosis was enhanced and the proliferation ability of TGF‐β‐activated CFs was increased after BPS combined with CAY (Figure 4C,D). The change trend of cAMP was similar to that of p‐CREB (Figure 4E). Furthermore, the relative ratio of p‐CREB/CREB showed that BPS treatment increased p‐CREB level in CFs, and the antagonist CAY could reverse this effect (Figure 4F). In addition, CAY10441 could down‐regulate the expression of GSK‐3β induced by BPS (Figure 4F). These results suggest that BPS can up‐regulate p‐CREB and cAMP, and eventually induce the inhibition of myocardial fibrosis.

## 4 DISCUSSION

After MI, cardiomyocytes are damaged or even apoptosis due to hypoxia, and myocardial remodeling leads to heart failure, which affects cardiac function and leads to the restriction of life activities and even death.^23^ Myocardial fibrosis is the main cause of the progression of most cardiovascular diseases, usually resulting in structural changes in the myocardium and vessel walls.^24^ Therefore, understanding the pathogenesis of myocardial fibrosis after MI can provide research directions and therapeutic targets for MI prevention and treatment. This study can provide more theoretical basis for the application of BPS in the clinical treatment of myocardial fibrosis after MI.

Ligation of the left anterior descending coronary artery in mice is a common model for studying postinfarcted ventricular remodeling and heart failure. The pathological changes of the heart after myocardial infarction can well simulate the pathological manifestations of human myocardial infarction.^25^, ^26^ Myocardial fibrosis produces large amounts of α‐SMA and collagenⅠ, which increase muscle tissue stiffness and decrease cardiac contractility, leading to fatal arrhythmia.^27^ BPS is a stable prostacyclin analog, with vasodilatation, antiplatelet agglutination, anti‐inflammatory, and other pharmacological effects.^28^ This study is the first to identify GSK‐3β as a target of BPS. BPS improved myocardial fibrosis and upregulated GSK‐3β protein expression in male SD rats after the operation. BPS can down‐regulate α‐SMA level and up‐regulate GSK‐3β protein expression after TGF‐β stimulation of CFs, which is consistent with the in vivo experimental results. Furthermore, GSK‐3β knockdown can reverse the effect of BPS on TGF‐β‐activated CFs, enhance α‐SMA expression, and promote CFs proliferation.

Niwano and colleagues have reported that BPS upregulated endothelial nitric oxide synthase (eNOS) gene expression through enhanced eNOS promoter activity in endothelial cells.^29^ Lian and colleagues suggested that BPS can increase induced nitrogen monoxide synthase (iNOS) expression by inducing cAMP response elements (CRE) binding to the iNOS promoter in cardiomyocytes.^30^ Notably, one study reported that YBX1 binds to the promoter of GSK‐3β and induces GSK‐3β expression to promote the growth of pancreatic cancer cells.^31^ YBX1 played a key role in cardiomyocyte proliferation, which was inferred to be an essential regulator of cardiac regeneration.^32^ At the same time, studies have found that prostaglandin E2 (PGE2), which is a prostaglandin series drug with BPS, can enhance HCC cell invasion by upregulating YBX1 expression.^33^ Based on the above studies, we consider whether BPS regulates the expression of GSK‐3β by promoting the binding of GSK‐3β promoter to YBX1. Luciferase and ChIP assay in this study confirmed that BPS could regulate GSK‐3β expression by promoting the binding of GSK‐3β promoter to YBX1.

Studies have shown that prostaglandin analog ACT‐333679 can activate cAMP signaling in pulmonary fibrosis, and the activation of intracellular cAMP signaling CFs can promote the antifibrotic effect.^34^, ^35^, ^36^ Knockdown of the prostaglandin receptor caused severe myocardial fibrosis and downregulation of p‐CREB and cAMP.^37^ After treatment in rats with MI, the intracellular cAMP and p‐CREB concentrations were upregulated.^38^ It is worth noting that BPS was found to be involved in cAMP signaling activation in CFs.^39^ In this study, BPS induced upregulation of p‐CREB and cAMP, resulting in reduced fibrosis, which was reversed by GSK‐3β knockdown or IPR antagonists.

In conclusion, after BPS treatment, the binding of YBX1 to the GSK‐3β promoter was increased, and the protein expression of GSK‐3β was upregulated, which further caused the upregulation of p‐CREB and cAMP expression, and finally caused the downregulation of fibrosis level (Figure schematic model). This study illustrates the relationship between BPS and myocardial fibrosis after MI to a certain extent, and opens a new horizon for intervention of myocardial fibrosis.

## AUTHOR CONTRIBUTIONS

**Xing‐Xing Li**: Conception; writing, review. **Yun‐Zhe Wang**: Design experiments and review. **Chuang Liu**: Analysis of data and review. **Guo‐Wei Fu**: Methodology and review. **Jun Li**: Methodology and review. **Jin‐Ying Zhan**g: Conception; writing and review.

## CONFLICT OF INTEREST STATEMENT

The authors declare no conflict of interest.

## ETHICS STATEMENT

All animal experiments are approved by the Ethics committee of The First Affiliated Hospital, Zhengzhou University.

## Supporting information


**Supplementary information**.

## Data Availability

All data generated during this study are available within the article.
