# Supplementary figures and images for: Beraprost sodium attenuates the development of myocardial fibrosis after myocardial infarction by regulating GSK‐3β expression in rats
Source: Immun Inflamm Dis. 2023 Nov 9;11(11):e1050. doi: 10.1002/iid3.1050 (PMC10633815; doi:10.1002/iid3.1050)

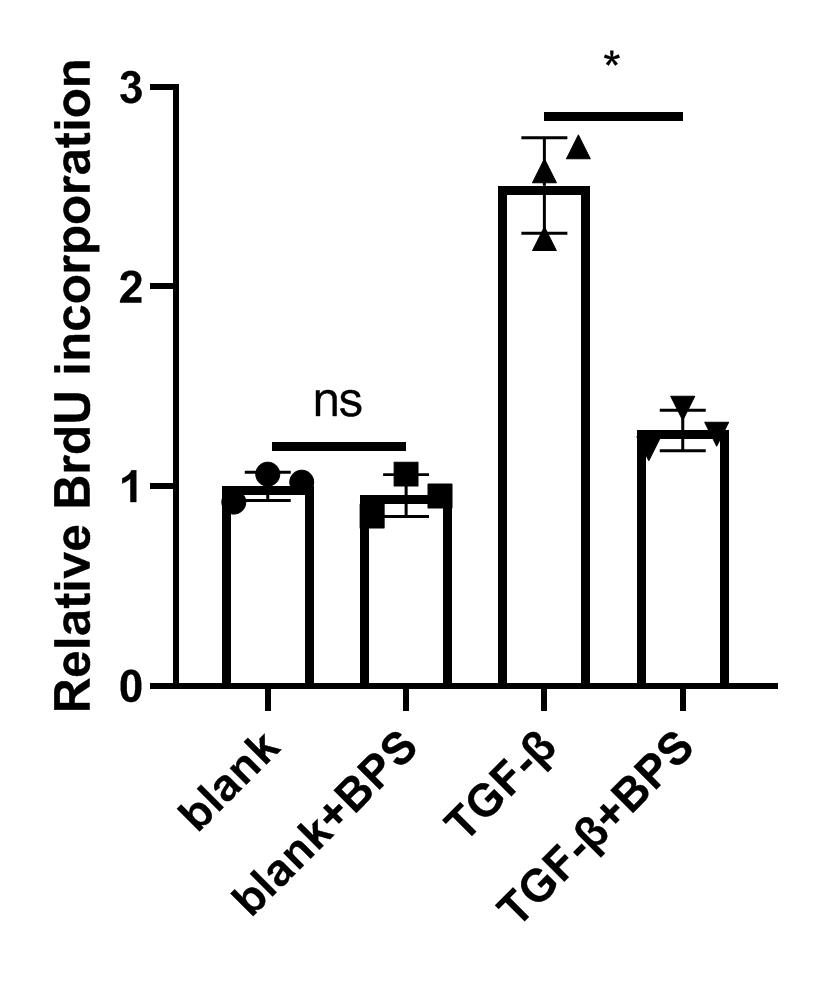

Supplement: Supplementary file 1 — Supplementary information. [file IID3-11-e1050-s001.jpg]
